# Platinum-Based Nanoformulations for Glioblastoma Treatment: The Resurgence of Platinum Drugs?

**DOI:** 10.3390/nano13101619

**Published:** 2023-05-12

**Authors:** Paula Alfonso-Triguero, Julia Lorenzo, Ana Paula Candiota, Carles Arús, Daniel Ruiz-Molina, Fernando Novio

**Affiliations:** 1 Institut de Biotecnologia i de Biomedicina, Departament de Bioquimica i Biologia Molecular, Universitat Autònoma de Barcelona, 08193 Cerdanyola del Vallès, Spain; paula.alfonso@icn2.cat (P.A.-T.); julia.lorenzo@uab.es (J.L.); anapaula.candiota@uab.cat (A.P.C.); carles.arus@uab.es (C.A.); 2 Catalan Institute of Nanoscience and Nanotechnology (ICN2), CSIC and BIST, Campus UAB, Bellaterra, 08193 Barcelona, Spain; dani.ruiz@icn2.cat; 3 Departament de Bioquímica i Biologia Molecular, Universitat Autònoma de Barcelona, 08193 Cerdanyola del Vallès, Spain; 4 Centro de Investigación Biomédica en Red, Bioingeniería, Biomateriales y Nanomedicina (CIBER-BBN), 08193 Cerdanyola del Vallès, Spain; 5 Departament de Química, Universitat Autònoma de Barcelona (UAB), Campus UAB, 08193 Cerdanyola del Vallès, Spain

**Keywords:** glioblastoma, platinum drugs, nanoformulation, chemotherapy, brain tumours

## Abstract

Current therapies for treating Glioblastoma (GB), and brain tumours in general, are inefficient and represent numerous challenges. In addition to surgical resection, chemotherapy and radiotherapy are presently used as standards of care. However, treated patients still face a dismal prognosis with a median survival below 15–18 months. Temozolomide (TMZ) is the main chemotherapeutic agent administered; however, intrinsic or acquired resistance to TMZ contributes to the limited efficacy of this drug. To circumvent the current drawbacks in GB treatment, a large number of classical and non-classical platinum complexes have been prepared and tested for anticancer activity, especially platinum (IV)-based prodrugs. Platinum complexes, used as alkylating agents in the anticancer chemotherapy of some malignancies, are though often associated with severe systemic toxicity (i.e., neurotoxicity), especially after long-term treatments. The objective of the current developments is to produce novel nanoformulations with improved lipophilicity and passive diffusion, promoting intracellular accumulation, while reducing toxicity and optimizing the concomitant treatment of chemo-/radiotherapy. Moreover, the blood–brain barrier (BBB) prevents the access of the drugs to the brain and accumulation in tumour cells, so it represents a key challenge for GB management. The development of novel nanomedicines with the ability to (i) encapsulate Pt-based drugs and pro-drugs, (ii) cross the BBB, and (iii) specifically target cancer cells represents a promising approach to increase the therapeutic effect of the anticancer drugs and reduce undesired side effects. In this review, a critical discussion is presented concerning different families of nanoparticles able to encapsulate platinum anticancer drugs and their application for GB treatment, emphasizing their potential for increasing the effectiveness of platinum-based drugs.

## 1. Introduction

Glioma is a general term used to describe primary brain tumours with an imbalance between high mortality and morbidity compared to their low incidence. These tumours are classified according to their originating cell (supportive glial cells) including astrocytomas, oligodendrogliomas, ependymomas, and mixed gliomas. Glioblastomas (GB) are the most malignant and frequent type of primary astrocytomas, accounting for about 33% of all brain tumours and about 80% of the total malignant central nervous system (CNS) tumours in adults [1]. Despite several efforts to improve GB treatment, nowadays it is still a deadly disease with poor prognosis and a median survival of less than 2 years upon diagnosis and a 5-year survival rate of 5.1% [2]. Moreover, GB is characterized by a diffuse infiltration of the adjacent brain parenchyma and development of drug resistance to standard treatments [3].

In addition to GB tumour cells, it is important to consider the tumour microenvironment (TME) factors in which neoplastic and non-neoplastic (e.g., tumour-associated macrophages, infiltrating lymphocytes) cell types interact, influencing GB growth, progression, and therapy resistance [4,5]. Recent studies indicate that differentiated tumour cells may dedifferentiate, acquiring a stem-like phenotype, in response to microenvironment stressful conditions such as hypoxia, acidic extracellular pH, or presence of nitric oxide [6]. Moreover, patient habits such as smoking also can induce pharmacokinetic interactions, through prevention of proapoptotic events or downregulation of proapoptotic proteins, and have a great impact on the effectiveness and toxicity of anticancer drugs [7]. As a result, adjustments of drug dosages should be taken into account in specific cases.

The standard of care for many years has consisted of surgical resection followed by radiotherapy (RT) and concomitant chemotherapy (CT), first described by Stupp et al. [8]. However, due to the location and infiltrative nature of GB, complete surgical resection of the tumour is often not feasible without a high risk of neurological damage for the patient [9]. The main anticancer drug used for GB treatment is temozolomide (TMZ), although acquired resistance of TMZ often contributes to the poor efficacy of the treatment, and patient survival is only slightly improved in few months. After almost two decades since the initial publication, and several efforts devoted in preclinical and clinical studies, the harsh truth is that the outcome of GB therapeutic approaches has not significantly improved.

Thus, the failure in GB treatment by now is attributable to different challenges during therapy, and also to interactions among different cells within the tumour microenvironment (TME). Therefore, methods to monitor genomic heterogeneity and even immune evasion would be interesting to adequate the therapy, using non-invasive strategies, to improve delivery across the blood–brain barrier and facilitate the success of personalized therapies [10]. On top of that, the economic burden of GB treatment has been reviewed in terms of cost-of-illness and cost-effectiveness studies [11].

## 2. Current Landscape for GB Treatment

The most effective standard clinical protocol for GB used nowadays was established back in 2005 [8] and, as previously stated, involves maximum surgical resection without compromising neurological functions followed by a combination of CT (oral administration of TMZ) and RT, and a period of adjuvant TMZ. However, there are several challenges related to treatment effectiveness: the infiltrative GB nature hampers complete tumour removal, since tumour excessive resection can lead to brain dysfunction, while incomplete removal may lead to tumour regrowth from remaining tumour cells. Moreover, a key point to brain tumour treatment is the contribution of the biological barriers, blood–brain barrier (BBB) and blood–tumour barrier (BTB) to restrictions in local drug delivery.

TMZ, approved by the FDA in 1999, is the most commonly used anticancer drug for GB treatment due to its lipophilic nature enabling it to easily cross the BBB [12]. Although TMZ demonstrated increasing patient survival and suppressing tumour growth in early stages of disease, ca. 50% of treated patients do not respond to this anticancer drug. Additionally, several GB cell lines such as U87, U251, U373, or T98G have been shown to develop TMZ resistance [13,14,15]. The combination of TMZ with radiotherapy does not significantly improve GB patient survival [16]. Then, even after aggressive therapy, overall survival is below 2 years and therapeutic alternatives are rather limited since surgery is not always feasible [17]. Approaches such as tumour-treating fields (TTFields), in which alternating electrical fields are delivered via cutaneous transducer arrays, inhibited GB cell proliferation by interfering with mitotic apparatus, although a recent review highlighted that other effects related to TTFields such as immunogenic signaling or anti-migratory effects can be an added value in GB treatment [18].

Alternative chemotherapeutic approaches offered as second-line treatment upon TMZ failure include nitrosoureas such as lomustine, carmustine, fotemustine [19], and antiangiogenic agents which are human monoclonal antibodies that inhibits vascular endothelial growth factor (VEGF), such as Bevacizumab [20]. Additionally, many research efforts have been described towards targeted therapies in GB: targeting epidermal growth factor receptor (EGFR) [21], inhibiting protein kinase C (PKC) [22], or inhibiting phosphatidylinositol 3-kinase (PI3K) and mammalian target of rapamycin (mTOR) signaling pathways PI3K and mTor [23], respectively, among others. Considering the relevant immunosuppressive effects on the GB microenvironment and crosstalk with TME, immunotherapeutic approaches are also being currently exploited, alone or in combination with standard/novel GB therapies. These therapeutic actions have been basically focused on immune checkpoint inhibitors such as anti-PD-1 and anti-CTLA-4 or dendritic cell-based vaccine therapies, as well as adoptive T-cell therapies [24]. Interestingly, Liu et al. reported the co-encapsulation of TMZ and an immunity activator (OTX015), using ApoE decorated red blood cell membrane, to obtain a biomimetic nanomedicine. The resulting nanoformulation showed a brain-targeted drug co-delivery and synergistic chemoimmunotherapy in vivo, with marked tumour inhibition and enhanced anti-tumour immune responses [25]. However, the strong immunosuppressive environment in GB may require further additional strategies to sensitize GB cells to immunotherapy [26]. Altogether, these factors contribute to the lack of a cure for GB, requiring continuous research efforts towards novel treatment strategies.

An additional key factor to take into account is the side effects implication of the chemotherapeutics, and the interaction with concurrent medications. Although TMZ is one of the main chemotherapeutics used for glioblastoma first-line treatment, the administration of intense doses used in the clinical protocol provoke relevant side effects such as bone marrow problems (leading to the need of treatment halting due to anemia, thrombocytopenia, etc.) [27]. On the other hand, side effects of nitrosoureas are related to bone marrow, as well as liver, kidney, and lung damage, and an increased risk of developing leukemia [28]. In addition, nitrosoureas can cross the blood–brain barrier and cause neurotoxicity. Platinum complexes, and more specifically cisplatin-related therapeutics, are usually associated with nephrotoxicity and neurotoxicity [29]. Any type of therapy directed towards fast-proliferating cells might affect cell populations such as digestive system, skin, and immune cells, among others. Some therapeutic combinations have demonstrated to be effective and act synergistically for GB treatment. Moreover, since combination drugs target multiple pathways, their administration can cause significant savings: smaller drug doses, lower treatment failure rate, and slower development of drug resistance. However, it is important to consider some limitations to the use combination therapy in GB since it can trigger cumulative side effects and modest clinical benefit [30]. In this scenario, it is important to build mathematical models of synergism/antagonism of drugs and pathways for the prediction of drug combinations for the treatment of heterogeneous tumours in GB. It is also important to identifying synergistic interactions between chemotherapy, radiotherapy, and immunotherapy in order to maximize the antitumour potential of individual treatment approaches.

## 3. Platinum and Glioblastoma: State-of-the-Art

**Figure 1:**
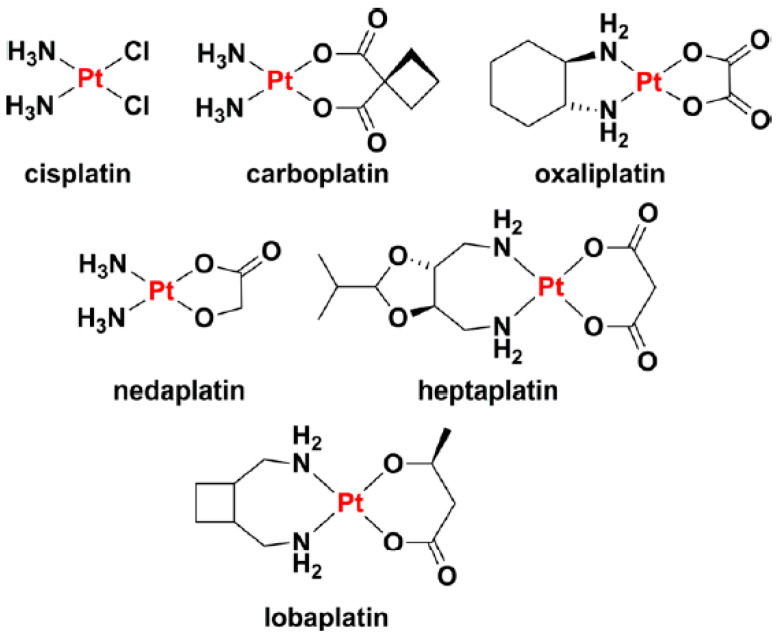
Structured of Clinically Approved Platinum Anticancer Drugs. Reproduced from Ref. [37] with permission of the copyright holder.

Platinum (Pt) complexes arise as an alternative option for treating brain tumours through chemotherapy. Platinum complexes are alkylating-like drugs that crosslink with the DNA, interfering with its repairing mechanism and inducing DNA damage and cell apoptosis [31]. Pt derivatives may also modulate anti-tumour immunity [32], impair tumour invasiveness through matrix metalloproteinase (MMP) downregulation, exhibit anti-angiogenic effects, and modulate MGMT DNA repair enzyme, among others. Pt-based drugs and prodrugs have demonstrated to be effective as anticancer agents in a variety of carcinomas (i.e., testicular, ovarian, lung, and head and neck cancer) [33]. The gold-standard Pt-based anticancer drugs cisplatin, carboplatin, and oxaliplatin have been proved to exert clinical effects in brain tumour patients [29,34,35,36]. Three additional platinum complexes (nedaplatin, lobaplatin, and heptaplatin) have been approved in specific countries. Figure 1 depicts the chemical structures of these approved Pt drugs [37]. This section may be divided by subheadings. It should provide a concise and precise description of the experimental results, their interpretation, as well as the experimental conclusions that can be drawn.

Pt complexes are described as effective therapeutic agents against gliomas [38,39,40] showing the following: (I) anti-angiogenic effects [41], (II) enhanced adjuvant therapy efficacy (TMZ and radiotherapy) [36,42,43], (III) successful combination with a tyrosine kinase inhibitor [44], and (IV) acceptable toxicity in chemotherapy-naïve patients with recurrent GB [45]. Moreover, the well know chemistry of Pt allows designing and generating an enormous variety of platinum-based complexes with stimuli-responsiveness properties in front of pH variation, redox activity, temperature changes, light irradiation, or enzyme overexpression [46]. These developments offer a good opportunity for obtaining site-specific prodrugs to maximize the therapeutic efficacy and minimize the side effect of platinum metallodrugs in anticancer therapies. However, reports in the literature describing the use of Pt, either alone or in combination in different approaches/scenarios, have controversial results. In vitro studies with human SNB19 and U87 GB cells reported the use of non-conventional Pt complexes (platinum-acridine hybrid agent) with excellent results and toxicity profiles superior to cisplatin [47]. Different therapeutic approaches have been tested—mostly in vitro—in combination with Pt such as bee venom [48], β-elemene extracted from curcuma wenyujin [49], nitrosoureas [50,51], bioconjugates combining EGFR targeting [52], microRNAs (miRNAs) [53,54], protein kinases C [55], tumour necrosis factor (TNF) [56], a histone deacetylase inhibitor (HDACi) [57], topoisomerase II [58], PI3K inhibitors [59], and a BK (large conductance, Ca^2+^-activated K^+^) channel inhibitor [60]. In addition, a combination of p(65)+Be neutrons irradiation plus cisplatin was attempted in U87 GB cells, resulting in a marked reinforcement of cytotoxic effect [61]. 

Regarding in vivo approaches, both in preclinical and clinical settings, single-agent carboplatin/cisplatin has been attempted as a ‘rescue’ treatment for high-grade glioma (HGG)-afflicted patients who did not respond after treatment with chemotherapy, nitrosoureas, or temozolomide, showing only discrete, non-significant improvement in outcome [62]. When coming to drug combination, a phase II trial combining TMZ and cisplatin was performed in pretreated and recurring HGG patients (GB and grade III gliomas treated with standard surgery + chemo-radiotherapy) [35]. This study reported a progression-free survival of 35% (at 6 months) and 13.8% (at 12 months), although grades 4 and 5 side effects were observed. Although results in this study were promising, the same authors recognized that they originated from a small, non-randomized cohort, and a more detailed studied would be needed to achieve confident conclusions in this regard. Pt complexes have also been explored in combination with other chemotherapy approaches, such as a dose-intense TMZ [63,64], both in preclinical models [65] and phase II trials [66]. Moreover, Rousseau et al. reported the combination of cisplatin (delivered via convection-enhanced delivery (CED)) with photon irradiation leading to enhanced survival of F98 glioma-bearing rats [67]. Thus, Pt drugs may have great therapeutic potential when effectively delivered to the tumour regions [68]. Despite the favorable results previously described, in practice, Pt therapy is considered fourth-line chemotherapeutics, i.e., it will only be used when all standard therapeutic protocols fail. At this point, we are probably facing unfavorable scenarios, dealing with highly resistant tumours. The performance observed in clinical settings has limited incidence on the overall survival of patients with brain tumours [69], neither alone nor in combination with radiotherapy [70,71] or chemotherapeutics such as carmustine [71] and TMZ [72].

Despite initial benefits reported by some authors, the administration of such Pt(II) complexes is often associated with severe systemic toxicity resulting from long-term treatment [73]. Moreover, the BBB contributes to the scarce drug arrival to the brain when drugs are administered orally or intravenously (i.v.). This is a key factor to explain the poor activity of such complexes in brain tumours, since passive diffusion is uncommon, and it is only feasible for small lipophilic compounds or endogenous molecules that pass through specific transporters. In fact, the use of receptor-mediated transport methodologies has been studied for many years as the most efficient mechanism for drug delivery to the brain [74]. Different methods have been explored to minimize the BBB-related challenges and to overcome this limited uptake of drugs in brain tumour while minimizing side effects. These strategies include approaches to increase the BBB permeability [75,76], use of biodegradable implants directly in the tumour [77,78], or more aggressive approaches, such as the use of a catheter to deliver drugs directly into the brain through CED [79]. 

Despite successful results, the development of formulations that need local device placements for drug delivery are not attractive from a clinical point of view, so new approaches ensuring proper and efficient chemotherapeutic delivery still represent a major challenge nowadays [37]. The use of nanoparticles (NPs) to increase the i.v. local delivery of drugs into the brain is one of the most promising approaches [80,81]. Their small size and large surface area are suitable to increase the solubility and bioavailability of the anticancer drugs, facilitating BBB diffusion and concentrating higher CT doses in the brain parenchyma [82]. Although NPs can benefit from the enhanced permeability and retention (EPR) effect to access and be retained in tumour tissues, the possibility of their surface functionalization enables targeting receptors or specific (bio)molecules is an added value to increase specificity or to evade the mononuclear phagocyte system [83,84,85]. Additionally, NPs can transport different therapeutic agents at the same time, protecting them from premature metabolism/degradation and inducing a precise control of the drug release [86]. All these advantages convert nanoparticles into suitable systems to maximize therapeutic efficacy of anticancer drugs while reducing their undesirable toxic effects [87].

Specifically, for minimizing drug resistance and side effects of platinum-based anticancer drugs/prodrugs while increasing their efficacy, efficient delivery systems based on cancer-specific targeting can be used [88,89]. Thus, different drug delivery systems including liposomes [90], dendrimers [52,91], polymers [92], nanotubes [93], or inorganic [94,95] and hybrid [96] nanoparticles have been evaluated with these purposes. Among them, liposomes are one of the most developed and promising drug carriers for platinum-based drugs [97], showing excellent effects without toxicity in phase I clinical trials [98]. It has been demonstrated that the nanoformulation of platinum-based drugs can significantly reduce the drug toxicity [99], improving drug delivery to tumours with a concomitant reduction in glioma growth, without neurotoxicity, and showing increased survival rate [100]. Moreover, the combination of drug administration using nanoformulations with other methodologies to improve brain delivery such as the disruption of the BBB by non-invasive transient focused ultrasound methods has been described [101,102]. Due to the complexity and heterogeneity of tumours [103], it is suggested that each patient might need personalized and combined treatments to increase the efficacy of platinum-based anticancer drugs against brain tumours.

## 4. Platinum-Based Drugs Limitations and Opportunities

Many efforts have been made to improve brain tumour treatment efficacy. The clinical treatment using platinum-based anticancer drugs reveals a series of limitations related to their clinical application and to the signaling mechanisms that cause drug resistance [29]. However, different studies have demonstrated that the therapeutic efficacy of platinum-based anticancer drugs can be significantly higher than TMZ efficacy. Thus, some questions arise, namely (i) what are the specific limitations related to the use of cisplatin in the clinical/preclinical practice of brain cancer treatment, and (ii) can these limitations be minimized? Below we will comment on some of them.

### 4.1. Factors Related to Brain Uptake

Less than 5% of drug plasma concentration is detected in the brain after i.v. delivery of platinum-based drugs to nonhuman primates, due to high selectivity of the BBB [36]. Even in the case of malignant brain tumours with compromised BBB integrity, only modest platinum compounds uptake was reported [104]. On the other hand, it has been demonstrated that chronic treatment with platinum-based drugs results in platinum accumulation in the brain. Different studies reveal that most of the adverse effects of chemotherapy are cumulative and occur after chronic exposure to the drug [105]. Along with cumulative chronic side effects, platinum drug treatment (i.e., oxaliplatin) also causes transient acute neuropathy, affecting sensory nerves in particular [106].

### 4.2. Narrow Therapeutic Window

Cisplatin is a potent chemotherapeutic agent with good results for standard medulloblastoma treatment but not for GB therapeutic protocols [107,108]. However, there is no clear explanation for the differences observed in the clinical efficacy against medulloblastomas and GB, even though cisplatin is effective in vitro against the latter. Recent results demonstrate that treatment with intratumoural cisplatin may be an efficient approach for brain tumour treatment with an effective narrow therapeutic window [109].

### 4.3. Inappropriate Dosing Schedule

Frequency of administration may be a key issue when balancing effects against tumour cells and effects over host immune system. Platinum-based chemotherapy can enhance host antitumour immune responses in several ways, e.g., inducing immunogenic cell damage (ICD), increasing the activity of tumour-killing immune cells, enhancing the sensitivity of tumour cells to immune checkpoint inhibitors [110], and increasing calreticulin and major histocompatibility complex (MHC I) expression in vivo [111]. With this in mind, unsuitable administration schedules, in addition to tumour killing, may also adversely affect the host immune system, leading to opposed effects. It has been shown that the immune-related effects of these chemotherapeutics can be dependent on the drug specificity, distribution, dosing, tumour model, and type of immunotherapy in case of drug combination [109].

### 4.4. Extensive Off-Target Toxicity

The use of platinum drugs for the treatment of GB has shown minimal success, in part due to the extensive off-target toxicities such as nephrotoxicity or neurotoxicity [112,113,114]. This is not an isolated event, considering that all large molecules and most of the small ones fail to reach the brain within the required therapeutic levels [115], even in GB showing disrupted BBB. Thus, achieving suitable therapeutic doses within brain tumours sometimes requires the use of adjuvants [116,117], which may also contribute to severe adverse effects. Strategies considered for increasing BBB permeability [118] include cisplatin delivery within biodegradable polymer implants into the tumour bed of patients [119] or bypassing the BBB via injection under CED [120]. However, since some of the aforementioned approaches are not feasible in clinical practice, the real improvement of half-life and toxicity is rather limited. Several studies indicate that free Pt-drugs at high doses induce lymphodepletion and may hinder rather than stimulate antitumour immune responses [121]. However, new emerging information points to the broad and multi-faceted therapeutic potential of platinum-based agents, improving the therapeutic ratio. Recent recognized immunomodulatory properties of platinum compounds seem to be able to overcome many of the mechanisms related to GB immune evasion [122]. The main advantages of nanoformulations are the site-specific accumulation in the brain tumour, minimizing the systemic toxicity from therapeutic drugs and reducing the off-target effects [123].

### 4.5. Sequestering/Deactivating Reactions

**Figure 2:**
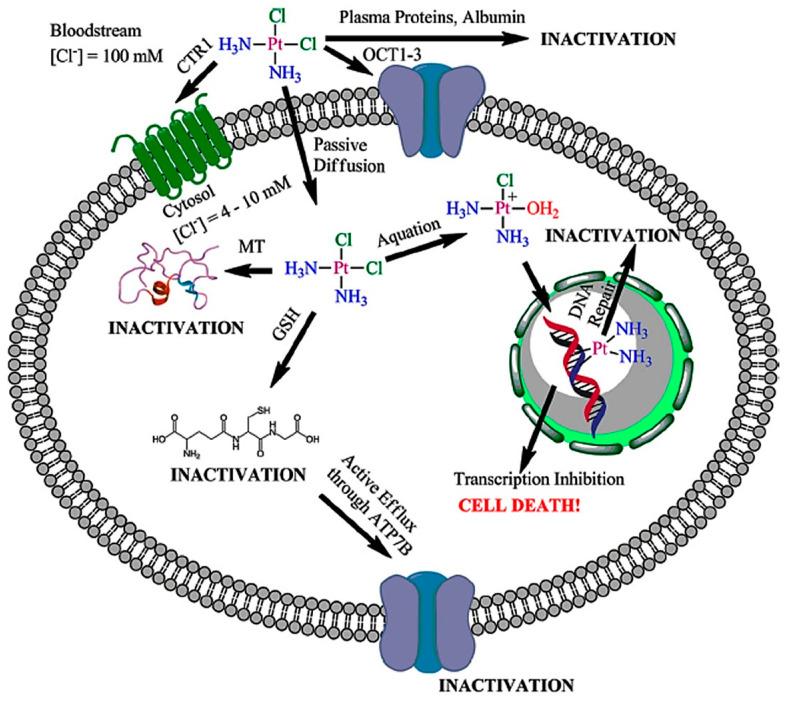
Deactivation and/or sequestration pathways of cisplatin. In extracellular environment, through interaction with serum albumin; once it is inside the cell, sequestration by coordination to metal-thioneins (MT) or inactivation through reaction with glutathione (GSH). This inactivation generates adducts removed from the cell through specific pumps such as ATP7B, implicated in cisplatin resistance. Reproduced from Ref. [124] with permission of the copyright holder.

One of the main objectives in platinum-based therapies is to minimize unwanted side reactions with biomolecules prior to DNA binding. For this purpose, the development of Pt(IV) prodrugs (most of them obtained by oxidation of the Pt(II)-related complex) afforded complexes with fine-tune desired biological properties such as lipophilicity, redox stability, cancer-cell targeting, improved cellular uptake, reduced off-target toxicity, and in some cases capability to modify the mechanism of action of the Pt(II) counterpart [37]. Reduction in the inner Pt(IV) center to anticancer-active Pt(II) due to the intracellular reductive environment, in concert with the loss of two ligands, is thought to be essential for the anticancer activity of these agents. Moreover, the possibility of attaching additional ligands to the octahedral coordinative sphere of the Pt(IV) metal center allows modifying its physicochemical properties and also facilitates attachment to nanoparticles and other carrier systems. The use of Pt(IV) complexes also offers solutions to different deactivation/sequestration pathways that can reduce the therapeutic actuation of platinum complexes, preventing cancer cells from triggering apoptosis and inducing the platinum complexes’ resistance (see Figure 2) [124].

Apart from opportunities offered by the nanoformulations, the unique properties of drug delivery vehicles can additionally provide capabilities of in vivo tracking of encapsulated drugs [125]. Nanoparticles have demonstrated potential as dual imaging contrast agents for magnetic resonance imaging (MRI) and computed tomography (CT) in glioma diagnosis [126]. Considering the disappointing therapeutic landscape for GB patients, discovering novel and safe methods to encapsulate and enhance drug delivery overpassing the BBB while improving biodistribution/chemical stability and decreasing side effects, has overall become a medical priority.

## 5. Platinum-Based Drugs Limitations and Opportunities

The studies published to date concerning the use of platinum-based nanoformulations for GB treatment were grouped into different categories according to the type of drug used, as follows. A summary of the principal examples is shown in Table 1.

### 5.1. Cisplatin

Duan et al. summarized several cisplatin-based nanoformulations with potential for clinic translation [127]. Reports related to the use of NPs for brain tumour treatment date back at least 10 years, with the encapsulation of cisplatin in polymeric NPs [128,129,130,131], polymeric micelles [132,133,134], polymeric conjugates [135,136], dendrimers [137,138], liposomes [139,140,141], nanocapsules [142,143], or its integration into metallic nanoparticles [144,145,146,147], silica nanoparticles [148,149,150], or hybrid nanoparticles (i.e., carbon nanotubes, nanoscale coordination polymers) [96,151,152,153,154,155,156,157,158]. Most of the published examples showed improved antitumoural effects, enhanced BBB crossing, and decreased side effects in comparison with free drugs. These benefits are related to the intrinsic properties of the NPs that can avoid the drug systemic toxicity, modulate the release of the therapeutic agent, and increase its accumulation into the tumour area by passive or active targeting processes.

Different metal-based NPs containing cisplatin drugs designed for GB treatment were reported. Makharza et al. described a nanocarrier combining γ-Fe_2_O_3_ NPs with nanographene oxide (NGO) for the selective vectorization of cisplatin. While NGO conferred high loading capabilities for cisplatin, the magnetic properties of the nanoparticles provided their magnetic guidance for targeting and delivery of therapeutics. The combination resulted in a sustained in vitro drug release with therapeutic potential against human U87 GB cells with negligible toxicity, together with the possibility to spatially control the drug delivery in the site of action [159]. 

**Figure 3:**
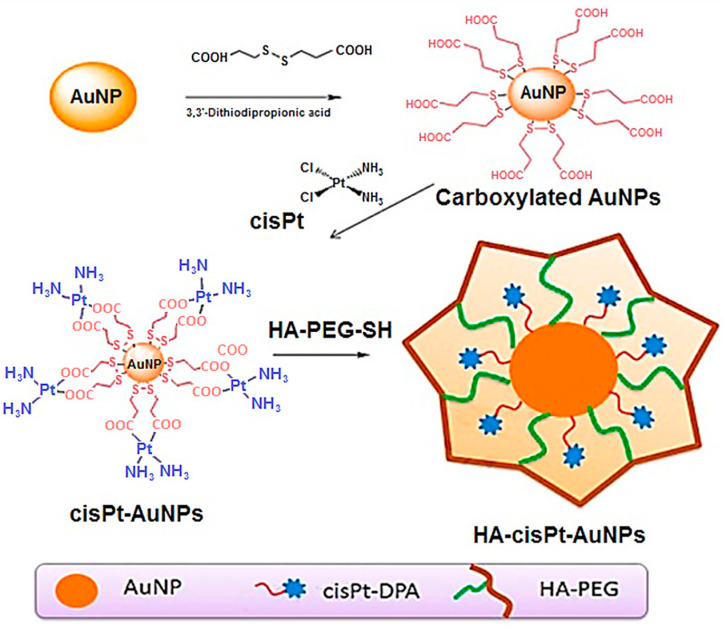
Preparation scheme of HA-CisPt-AuNp. AuNp: gold nanoparticle; CisPt: cisplatin or cis-diamminedichloroplatinum(II); DPA: 3,30-dithiodipropionic acid; HA: hyaluronic acid; and PEG: polyethylene glycol. Reproduced from Ref. [161] with permission of the copyright holder.

A couple of publications were reported in 2018 using gold nanoparticles (Au-NPs) for GB combined with radiation treatment. Coluccia et al. described that cisplatin conjugated Au-NPs chemotherapy was synergistic with radiation and MR-guided Focused Ultrasound (MRgFUS). Namely, viability assays with GB cell lines in vitro (U87, U251, T98G, U138) demonstrated cell growth inhibition compared to free cisplatin and showed marked synergy with radiation therapy, while in vivo studies showed increased BBB permeability and brain drug delivery through MRgFUS [160]. In another example, Gotov et al. also presented cisplatin conjugated Au-NPs coated with hyaluronic acid [161]. Similarly, NPs showed enhanced cytotoxicity activity in comparison with free cisplatin in human breast adenocarcinoma MCF-7 cells, human primary U87 GB cells, or murine fibroblast NIH/3T3 (control) cell lines (see Figure 3). In vivo antitumour efficacy was demonstrated in tumour models upon i.v. administration and subsequent exposure to a near infra-red laser in the tumour site. These drug-loaded NPs showed marked in vivo activity due to their (i) long time of circulation in blood stream and (ii) selective accumulation in the tumour. This formulation demonstrated an enhanced therapeutic effect and reduced toxicity of cisplatin combining chemotherapy and laser treatment.

In vivo experiments reported back in 2010 with the commercial agent Lipoplatin™, which had already reached phase II/III clinical studies for non-small-cell lung cancer (NSCLC) [162], HER2/neu negative metastatic breast tumour [163], and advanced gastric tumour, were not successful [164]. Unexpectedly, its administration using CED showed marked neurotoxicity resulting in death within a few days, while the i.v. administration was well tolerated. The same authors have also tested a home-made nanoformulation combining cisplatin with lipid cholesteryl hemisuccinate (CHEMS), with a Pt loading efficiency of 25%. After 24 h treatment, CHEMS showed higher in vitro cytotoxicity against F98 glioma cells in comparison with free cisplatin and an excellent intracerebral retention in F98 glioma-bearing rats. Unfortunately, 10–14 days after administration, CHEMS unveiled dose-dependent neuropathologic findings [165]. Nowadays, there is an intense effort to promote targeting upon NP surface functionalization with peptides, antibodies, or small molecules recognizing transporters, antigens, or receptors characteristic of tumoural cells [166]. Shein et al. described liposomes with sustained release of cisplatin reaching relevant intracellular concentration in U87 GB cells. This promising result was achieved thanks to the conjugation of liposomes with antibodies against the vascular endothelial growth factor (VEGF) and its receptor type II (VEGFR2), which account for resistance and rapid progression of brain tumours [167]. Later on, Ashrafzadeh et al. described pegylated liposomal formulations encapsulating cisplatin and decorated with thiolated OX26 monoclonal antibodies to target transferrin receptors (TR). These liposomes caused an increase in the cellular uptake by 1.4-fold in comparison to non-targeted analogous and an increase by 1.7-fold in the mean survival of the C6 glioma-bearing rats compared to non-targeted ones with notable reduction in toxicity effects [168].

Together with liposomes, polymeric NPs have been one of the most developed nanoformulations for cisplatin delivery in GB treatment. One of the first developments reported consisted in Pt-bearing Poly(Lactide-Co-Glycolide) (PLGA) NPs coated with protamine. These nanosystems were reported to cross the BBB using an in vitro model of bovine brain microvessel endothelial cell assay performed in 2014 [169]. In addition, the same study reported therapeutic activity against U87 human GB cells with these NPs. Similar poly(lactic-co-glycolic acid)-block-polyethyleneglycol NPs were used to target mitochondrial DNA in cisplatin-resistant cells. This was achieved due to their high encapsulation payloads and mitochondria-targeting abilities upon surface functionalization with a triphenylphosphonium cation [170]. More successful experiments were performed with biodegradable PLGA NPs in 2014, which provided cisplatin-controlled release, with brain penetration and subsequent tumour targeting observed in ex vivo human and murine brain samples [171]. Experiments reported by Shahmabadi et al. using cisplatin-loaded (25% encapsulation efficiency) polybutylcyanoacrylate (PBCA) nanoparticles layered with polysorbate 80 failed to cross the BBB, most likely due its diameter average—larger than 400 nm [172]. PEGylated-Poly(aspartic acid) NPs encapsulating cisplatin with deep tumour penetration after local administration by CED were described by Zhang et al. [173]. With these brain-penetrating NPs, cisplatin reached concentrations feasible to kill tumour cells without healthy brain toxicity. This was demonstrated in in vivo experiments with rats bearing F98 orthotopic gliomas whose median survival was significantly increased in comparison to animals treated with free cisplatin. Later on, in 2018, poly(ethylene oxide)-triblock polymers were combined with Gd^3+^/cisplatin to obtain micelles which can act as contrast agents in MRI acquisitions while increasing the therapeutic effect of free cisplatin. In fact, the formulated prodrug exhibited up to 50-fold increased accumulation in human GB cell lines and up to 32-fold enhanced subsequent Pt-DNA adduct formation in comparison with free cisplatin [174]. Moreover, in vivo MRI monitoring of Gd-bearing nanoparticles within the brain after CED determined their promising potential as multifunctional drug delivery systems for both therapy and MRI tracking. Other developments include nanogels, which are able to encapsulate cisplatin. Such approaches showed reduced toxicity in comparison with the free drug, inhibited tumour growth, and promoted extended survival of C6 glioma-bearing rats when the nanogel was functionalized with a monoclonal antibody targeted to connexin 43, a protein highly expressed in the tumour periphery of C6 gliomas [175].

Since GB usually relapses after eventual transient response, and there is no validated second-line treatment, the co-encapsulation of synergistic drugs was proposed as a therapeutic alternative. It has been shown that cisplatin and fisetin encapsulated into liposomes have a synergistic effect, due to the combination of the anti-angiogenic effect of fisetin with the cytotoxic effect of cisplatin. The formulation showed an additive effect of cisplatin and fisetin against GB cells, demonstrating antitumoural effect [176]. Although combinatorial therapy based on TMZ plus cisplatin showed promising potential for GB therapy in clinical trials, the limited BBB crossing, poor targeting of GB tissues/cells, and systemic toxicity altogether hindered its efficacy in GB therapy. More recently, camouflaged GB cell membrane and pH-sensitive biomimetic nanoparticles demonstrated efficiently co-loading TMZ and cisplatin, providing transport across the BBB, and specifically targeting GB [177]. With this nanoformulation, a controlled release of drug cargos was obtained. A potent anti-GB effect in vivo was observed after treatment of mice bearing orthotopic U87 or the drug-resistant U251R GB tumours. The average survival was increased in mice receiving the combined drug administration compared to the survival time for mice receiving single-drug loaded nanoparticles, without noticeable side effects.

Apart from the development of novel nanoformulations, the understanding of drug transport across the BBB remains limited and represents a significant challenge for HGG treatment. There is an urgent need for predictive in vitro models with realistic and dynamic blood–brain barrier vasculature features. Thus, different models have been attempted to simulate the brain tumour vasculature such as microfluidic devices emulating BBB using self-assembled endothelial cells, astrocytes, and pericytes using modular layer-by-layer assembly [178]. The objective of such approaches is to investigate the transport of targeted nanotherapeutics across the BBB and into GB cells. In the reported study, cisplatin-loaded nanoparticles decorated with GB-targeting motifs to improve tumour trafficking were evaluated in this in vitro platform. The obtained results were compared with transport across mouse brain capillaries using intravital imaging, validating the ability of the platform to properly model the in vivo BBB transport. These models represent a significant advance, enabling in-depth investigation of brain tumour vasculature and accelerating the development of targeted nanotherapeutics.

The nanoformulation of cisplatin has demonstrated to remarkably improve its pharmacokinetics, which is especially unfavorable in case of glioblastomas, mainly due to challenges posed by biological barriers such as the BBB. However, the pharmacokinetic profile greatly depends on the type of nanostructure being used. In general, the integration of platinum complexes into nanoparticles allows for larger blood circulation time, more efficient arrival to the tumour zone while decreasing the amount of dosage needed, as well as protecting drugs from degradation, which increases stability. An example is a work published by Yu et al. that describes the pharmacokinetics of cisplatin-loaded polymeric nanoparticles [179]. The studies in vivo demonstrated that the selected nanoparticles had a long blood circulation time, the platinum concentration remained up to 46-fold higher than that of mice receiving equivalent doses of cisplatin, and the platinum concentration ratio of NPs to free cisplatin in tumours (lung tumour) reached as high as 9.4. Moreover, NPs improve the safety and tolerance in vivo, and improves the anticancer efficacy in comparison to cisplatin. Facts certainly change when talking about glioblastomas with the associated challenges for tumour delivery across the BBB: in this case, still, improvements can be obtained as shown by Ashrafzadeh et al., who reported a targeted pegylated liposomal cisplatin able to cause an increase in the cellular uptake and in brain tumour by 1.43 and 1.7-fold, respectively [168]. The administration of the NPs showed enhanced efficacy and reduced toxicity for the treatment of brain tumour. In fact, the blood–drug concentration was increased in comparison to cisplatin and then maintained in the effective range in a sustained mode. In general, the nanoformulations increase drug efficacy and low toxicity, and provide various pharmacokinetic benefits such as lowered drug accumulation with chronic drug dosing, and minimal fluctuation of drug concentration in blood.

### 5.2. Carboplatin

Carboplatin has a lower nephrotoxicity and ototoxicity incidence than cisplatin, although it is less active mainly due to its lower cellular uptake [180], thus repeated administration cycles with a large number of doses are required to inhibit tumour growth [181]. Amongst the platinum complexes (carboplatin, cisplatin and oxaliplatin), carboplatin exhibited the lowest toxicity while providing the best survival benefits. In addition, studies inducing DNA damage by low-energy secondary electrons produced by radiation suggest that carboplatin is better than cisplatin as a radiosensitizer [182]. These studies indicate that carboplatin is the best candidate among the approved platinum drugs for treatment of human brain tumours.

**Figure 4:**
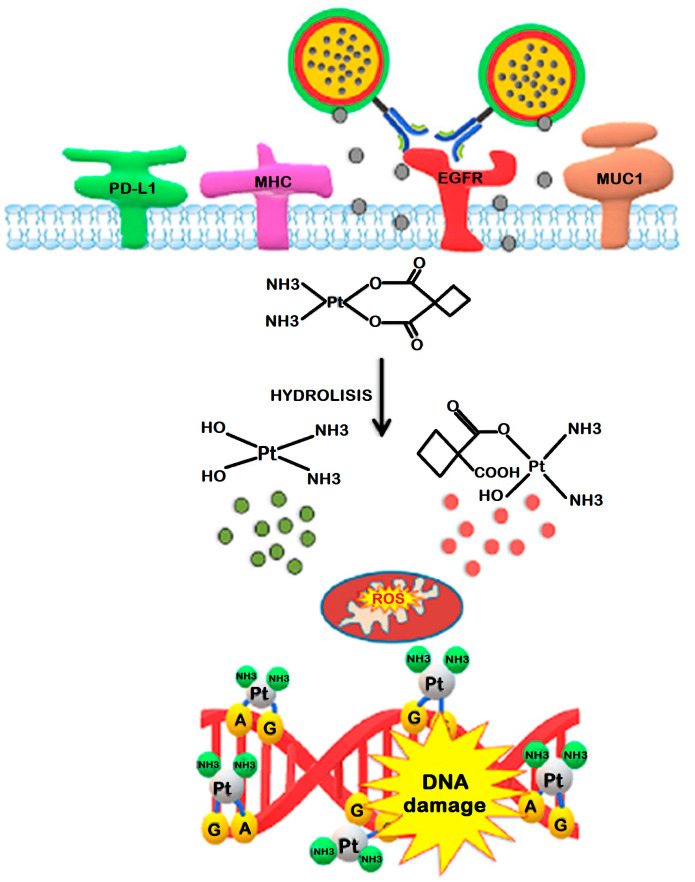
Schematic representation of targeted PBCA NPs conjugated with monoclonal antibodies against epidermal growth factor receptors (EGFR) and the platinum-based complexes drug delivery process. Reproduced from Ref. [187] with permission of the copyright holder.

In an earlier study was described the covalent coupling of carboplatin to N-(2-Hydroxypropyl)methacrylamide (HPMA) and the tetrapeptide glycyl-phenylalanyl-leucyl-glycine (GFLG), whose proteolytic cleavage allows for its intracellular drug delivery [183,184]. The resulting nanoparticles revealed a higher therapeutic effect in preclinical models (compared to free carboplatin) and were accepted in a Phase I clinical trial [185]. On the other hand, the encapsulation of carboplatin with poly (ε-caprolactone) NPs was able to minimize hemolysis and increase the cytotoxicity effect against U87 human GB cells, which was more remarkable than the free drug [186]. Recently, it has been reported that the use of carboplatin-loaded poly (butyl cyanoacrylate) (PBCA) NPs conjugated with monoclonal antibodies against epidermal growth factor receptors (EGFR) for GB treatment. In this study, both therapeutic efficacy and side effects of the resulting NPs were evaluated in vivo in a rat model of GB (see Figure 4) [187]. The findings showed a 40% augmented cytotoxicity compared to the free form of carboplatin. Moreover, in vivo studies demonstrated an increased survival and appearance of less side effects in brain, kidney, or liver, compared to rats treated with free carboplatin. In another study, authors have described biodegradable PLGA NPs with controlled carboplatin release in rat brain by CED [188]. The treatment involved recurrent infusions due to the rapid clearance of carboplatin from the brain, and improved outcome was observed in GB patients, essentially due to an increase in cytotoxicity against tumours, lower neuronal toxicity, and prolonged half-life.

Although liposomes are the principal nanosystems studied for drug delivery, the most appropriate liposomal formulation for CED brain tumour injection remains to be determined. Different liposomal carboplatin formulations were prepared and tested in vitro in F98 glioma cells and in Fischer rats carrying orthotopic F98 tumours. The results indicated that the therapeutic efficacy in vitro and in vivo may vary drastically depending on surface charge [189]. Cationic liposomes bind more efficiently to tumour cells, increasing their therapeutic efficacy in vitro although the median survival time did not significantly improve. Interestingly, anionic and pegylated liposomes diffuse better towards the tumoural area, increasing its concentration in the tumour, reducing its clearance rate, and reducing neurotoxicity relative to free carboplatin. Thus, intratumour injection of liposomal carboplatin nanoformulations is considered a promising alternative to the administration of pure carboplatin in the chemotherapeutic treatment of GB.

A comparative study tested cisplatin, oxaliplatin, carboplatin, Lipoplatin (liposomal formulation of cisplatin), and Lipoxal^TM^ (liposomal formulation of oxaliplatin) administered by intracarotid infusion to F98 gliomas orthotopically growing in Fischer rats. The nanoformulation of platinum compounds demonstrated reduced toxicity, improved cancer cell uptake, and increased survival rates either when combined or not with radiotherapy, in comparison with liposome-free drugs. Moreover, among the platinum compounds tested, liposomal carboplatin showed the largest survival increase when combined with radiation in vivo [97].

Interestingly, in the search of new routes of administration, Alex et al. encapsulated carboplatin into polycaprolactone NPs to target GB via the nasal route [190]. The NPs showed improved in vitro anti-tumour activity in comparison with the free drug against the human GB cell line LN229. Ex vivo permeation studies through sheep nasal mucosa showed a similar release pattern as for in vitro release studies. Moreover, in situ nasal perfusion administration in Wistar rats demonstrated that NPs show better nasal absorption than carboplatin solution while no severe damage to the integrity of nasal mucosa was detected. These results suggest that these nanoformulations are suitable to improve the nasal absorption of carboplatin and consequently to improve brain delivery.

### 5.3. Oxaliplatin

**Figure 5:**
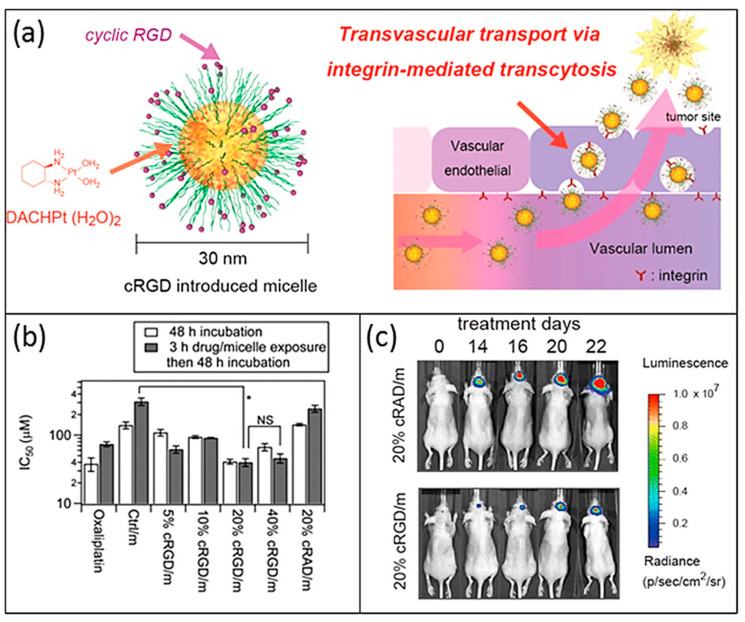
(**a**) Schematic showing the design strategy for the oxaliplatin encapsulation on the PEGl-glutamic acid (Glu) micelles functionalized with cyclic Arg-Gly-Asp (cRGD), and proposed transvascular transport for the NPs; (**b**) In vitro cytotoxicity, in U87 cells, of free oxaliplatin or resulting micelles with different percentages of targeting molecules (the data were analyzed using Student’s *t*-test, and * *p* < 0.001 was considered significant; NS: not significant); (**c**) Representative in vivo bioluminescence images of mice treated with 20% non-targeted and 20% of targeted micelles. Reproduced from Ref. [192] with permission of the copyright holder.

Oxaliplatin was demonstrated to induce multi-faceted anti-tumour effects, more effectively than cisplatin and carboplatin at drug concentrations/doses below those required to induce apoptosis, which fostered specific research in the GB area. However, in vivo preclinical studies were unsatisfactory since i.v. administration did not increase the median survival time of F98 glioma-bearing rats [104]. This may be most likely due to the BBB preventing drug accumulation in brain tumours, even when compromised such as in HGG [191]. To overcome these limitations, the drug was bound to PEG-glutamic acid (Glu) micelles functionalized with cyclic Arg-Gly-Asp (cRGD) in order to cross the BBB, target GB cells, and penetrate into GB via cRGD-mediated transvascular transport (see Figure 5) [192]. Their antitumour effect against GB was compared to the effect of control NPs functionalized with the nontargeted peptide. These nanoformulations exhibited significant growth inhibition effects against GB compared to the non-targeted micelles, as well as tumour accumulation, indicating the active transport of cRGD-mediated drug delivery across vascular and tumour barriers.

Later, the liposomal formulation containing oxaliplatin (Lipoxal™) was administered via CED, which successfully improved tumour accumulation and subsequent survival time in F98 glioma-bearing rats when combined with radiotherapy. Moreover, the maximum tolerated dose (MTD) of Lipoxal™ was 3-fold superior to that of free oxaliplatin [193]. More recently, You et al. reported multiwalled carbon nanotubes (MWCNTs) containing oxaliplatin functionalized with cell penetrating peptides (TAT) and the cancer targeting molecule biotin. This nanosystem enhanced oxaliplatin cytotoxicity towards glioma cells as a result of reactive oxygen species (ROS) overproduction while in vivo studies demonstrated its BBB penetration and outstanding antitumour efficacy against orthotopic glioma [194].

Apart from the gold-standard platinum complexes, there are some additional developments of novel platinum-based nanoformulations with interesting results concerning GB treatment. This is the case of a reported platinum-based polyethylenimine (PEI) polymer–drug conjugate with potential anti-GB stem cells. The cationic polymer presented a potent and specific toxicity in vitro. The results obtained from cytotoxicity studies with NCH421K and NCH644 GN cells indicated a necrotic cell death mechanism with an absence of apoptotic markers. Several markers also indicated that this cell death mechanism could induce an anti-cancer immune response [195].

### 5.4. Pt(IV) Prodrugs

Pt(IV) prodrugs have been used mainly to counteract cisplatin resistance and nephrotoxicity effects; while in its Pt(IV) oxidation state, side effects are considerably minimized, though inside the cells it is further reduced to the antitumoural active Pt(II) form [196]. Thanasupawat et al. reported self-assembled coiled nanotubes linked with a Pt(IV) complex that exhibit better in vitro and in vivo toxicity against human U87 GB cells (and cells obtained from human GB samples) than free Pt(IV), mostly due to the activation of multiple death pathways. Moreover, the nanosystems were active in subcutaneous/orthotopic U87 xenografts using intratumoural administration [93].

**Figure 6:**
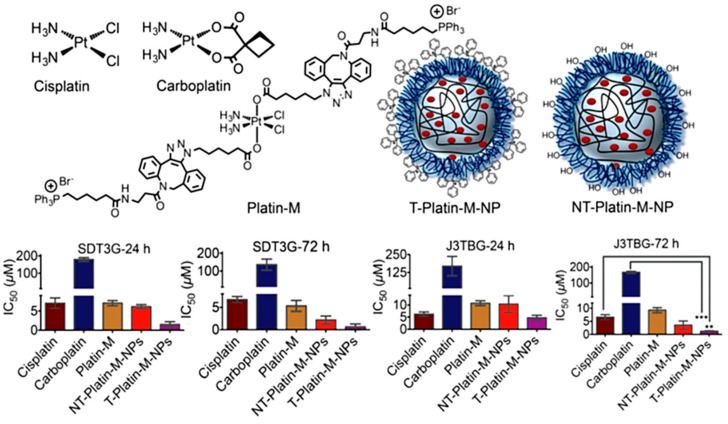
In vitro efficacy of T-Platin-M-NPs and comparison with cisplatin, carboplatin, Platin-M, and NT-Platin-M-NPs in two canine brain tumour cell lines, J3TBG and SDT3G. Cell viability was assessed by MTT assay after treatment for either 24 h followed by 48 h incubation or treatment for 72 h, depending on the drug concentration. The data are mean ± SD from three to five independent experiments. *** *p* < 0.001; ** *p* < 0.001–0.01. Reproduced from Ref. [128] with permission of the copyright holder.

A more complete study from Dhar and coworkers in 2015 reported a lipophilic polymeric NP carrying a Pt(IV)-prodrug (see Figure 6) with capabilities of mitochondria targeting [128]. The platinum prodrug of cisplatin (Platin-M) was encapsulated in targeted polymeric nanoparticles carrying the drug across the BBB and specifically towards mitochondria. NP-mediated controlled release of Platin-M and its subsequent reduction to cisplatin provoked the cross-linking with the mitochondrial DNA, thus forcing overactive cancer cells to undergo apoptosis. In vitro effects of the NPs in canine glioma and GB cell lines demonstrated to be much more effective than free cisplatin or carboplatin. In vivo biodistribution studies in healthy adult beagles after single i.v. injection showed high levels of Pt accumulation into the brain, with negligible amounts found in other organs. Moreover, no signs of neurotoxicity were observed, demonstrating the translational potential of these nanoformulation for applications in brain tumour treatment.

A recent study showed that NPs encapsulating an oxaliplatin prodrug and a cationic DNA intercalator, administered through CED, were able to inhibit the growth of TMZ-resistant cells from patient-derived xenografts, and hinder the progression of TMZ-resistant human GB tumours in mice, without causing any noticeable toxicity [197]. Such polymeric nanoformulations contain disulfide bonds which are cleaved in the reductive tumour environment, which affords a selective release of anticancer drugs. The reported research findings suggest that the administration of drugs with different mechanisms of action, combined with CED, may represent a translational strategy for the treatment of TMZ-resistant gliomas. Another example includes polyethylene glycol (PEG)-stabilized solid lipid nanoparticles (SLNs) containing different Pt(IV) prodrugs derived from kiteplatin. An in vitro BBB model of immortalized human cerebral microvascular endothelial cells (hCMEC/D3) was used for evaluating the ability of the SLNs to cross the BBB, while U87 human GB was used for cell internalization and antitumour efficacy studies. These nanoformulations demonstrated potent ability to permeate the in vitro BBB model with improved cellular uptake and higher reduction in cell viability in comparison with their free counterparts [198].

**Figure 7:**
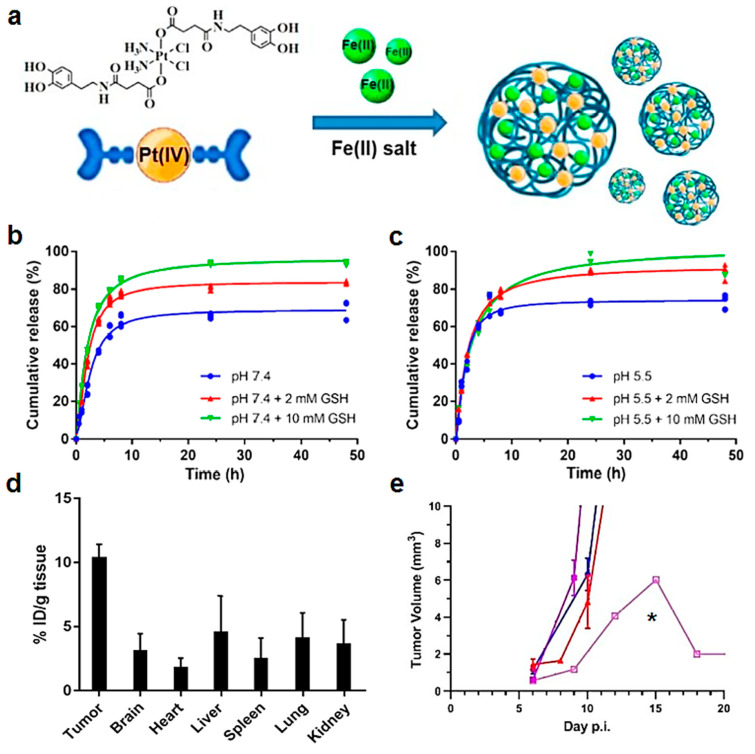
(**a**) Scheme of the Pt-Fe NCPs synthesis upon polymerization of a Pt(IV) prodrug with iron ions as metal nodes; Cumulative release profiles of Pt from Pt-Fe NCPs at 37 °C at (**b**) pH 7.4 and (**c**) pH 5.5 in PBS using the dialysis method in the absence or in the presence of glutathione (GSH); (**d**) Biodistribution of Pt-Fe NCPs in mice organs 1 h after administration (dosage of 1.5 mg/kg, n = 3); (**e**) Tumour volume evolution in the period 0–20 days post-implantation (p.i.) [blue: control; red: therapy starting point at day 10 p.i.; pink: therapy starting point at day 6 p.i.; *: case of cured mice with therapy starting point at day 6 p.i. Reproduced from Ref. [96] with permission of the copyright holder.

A novel type of nanoparticles containing Pt(IV) prodrugs as building block of nanostructured coordination polymers (NCPs) was recently described, with enormous potential for treating different cancers [157]. The versatility of coordination chemistry allows obtaining nanoparticles by reaction of a novel platinum (IV) prodrug and metal ions (i.e., iron or zinc) producing theranostic nanosystems [199]. Ruiz-Molina et al. designed a nanoparticle based on a coordination polymer with building blocks containing Pt(IV) complexes obtained from cisplatin and iron ions as metallic nodes. This nanoformulation presents dual pH and redox sensitivity in vitro, showing controlled release and comparable cytotoxicity to cisplatin against HeLa and GL261 GB cells. Interestingly, in vivo intranasal administration in orthotopic preclinical GL261 GB-bearing mice demonstrated increased accumulation of platinum in tumours, leading in certain cases to cure and prolonged survival of the tested cohort (Figure 7) [96]. Additionally, the presence of magnetically active iron(III) ions allows these NPs to be proposed as potential nanoprobes to be tracked in vivo by MRI, thus opening new opportunities for the design of intranasal glioblastoma therapies while minimizing side effects.

**Table 1:** Preclinical studies on the effect of nanoformulated anticancer Pt-complexes for GB treatment.

Pt Agent	Nanocarrier & Size	Form	Cells/Animal Models (Administration Mode)	Effect/Mechanism of Action	Ref.
Cisplatin	Iron oxide NPs in NGO (lateral width of 80–100 nm and thickness of 6.3 nm)	γ-Fe_2_O_3_ NPs coated by nanographene oxide (NGO) containing cisplatin	U87 cell line	Superparamagnetic-like behavior but showed little cytotoxicity at 10 μM.	[159]
Cisplatin	Au-NPs (7 nm)	Cisplatin conjugated to gold NPs	U251/U87 cell lines, U251 xenografts (intravenous injection)	Inhibited GB cells growth and showed marked synergy with MR-guided Focused Ultrasound (MRgFUS) therapy. In vivo assays demonstrated increased BBB permeability.	[160]
Cisplatin	Au-NPs (145 nm)	Cisplatinum attached to hyaluronic acid-functionalized gold NPs and coated with PEG	U87 cell line MCF-7 tumour-bearing mice (intravenous injection)	NPs inhibited GB cells growth and showed marked synergy with the thermal effect of applying NIR laser radiation.	[161]
Cisplatin	Liposomes (50–60 nm)	Lipoplatin^TM^ and novel cisplatin bound to coordination complexes with lipid cholesteryl hemisuccinate (CHEMS)	F98 glioma cell line F98 glioma-bearing rats (convection-enhanced delivery)	Potent in vitro cytotoxicity. High intracerebral retention after CED. CHEMS liposomes were better tolerated than Lipoplatin^TM^ and showed increased survival data in vivo.	[165]
Cisplatin	Liposomes (130–160 nm)	Cisplatin analogue and antibodies against VEGF or VEGFR2 conjugated on liposome surface	C6, U87 cell lines	Prolonged blood circulation time in C6 glioma-bearing rats. In vitro data confirmed that conjugation with specific antibodies increases the intracellular concentration of the liposomes and improves cytotoxicity.	[167]
Cisplatin	Liposomes (160 nm)	Liposomes decorated with OX26 monoclonal antibodies	C6 cell line C6 glioma-bearing rats (stereotactic injection)	Increased cellular uptake and increased mean survival time in vivo with notable reduction in toxicity effects.	[168]
Cisplatin	Polymeric NPs (105 nm)	Protamine-functionalized PLGA NPs	U87 cell line	Increased cellular uptake and cytotoxicity in vitro and ability to cross in vitro BBB model, improving therapeutic index.	[169]
Cisplatin	Polymeric NPs (50–145 nm)	PLGA-PEG copolymer NPs functionalized with a triphenylphosphonium cation	C6 cell line C6 glioma-bearing rats (intravenous injection)	Nanoparticles below 100 nm diffused within mice brain if densely coated with PEG.	[170]
Cisplatin	Polymeric NPs (114 nm)	PLGA NPs	U87 cell line	Significantly higher cytotoxicity than cisplatin, enhanced cellular uptake.	[171]
Cisplatin	Polymeric NPs (490 nm)	PBCA NPs	C6 cell line C6 glioma-bearing rats (intraperitoneal injection)	Similar cytotoxicity in comparison to cisplatin. Slightly longer mean survival time and reduced side effects.	[172]
Cisplatin	Polymeric NPs (70 nm)	PEGylated-Poly(aspartic acid) NPs	9L gliosarcoma and F98 glioma lines, orthotopic F98 brain tumour model (convection-enhanced delivery)	Reduction in toxicity associated with cisplatin and increased average survival in vivo.	[173]
Cisplatin	Polymeric NPs (9 nm)	Gd-grafted PEO micelles	U87/U251 cell lines, U87 orthotopic xenografts (stereotactic injection)	Hyperintense signal on T2-weighted MRI, 50-fold increased cellular accumulation, and 32-fold Pt-DNA adduct compared to free cisplatin.	[174]
Cisplatin	Nanogel (110 nm)	PEG-b-PMAA nanogels functionalized with monoclonal antibody to Cx43	C6 cells C6 glioma-bearing rats (intravenous injection)	Targeted delivery to C6 glioma. Prolonged the mean survival time of glioma-bearing rats.	[175]
Cisplatin + Glutathione Peroxidase	Iron NPs (80–125 nm)	Cisplatin and small interfering RNA (siRNA) targeting glutathione peroxidase attached to IONPs and functionalized with folic acid	U87 cell line, primary glioblastoma cell line (P3#GB), normal human astrocytes (NHAs) U87orthotopic xenografts (stereotactic injection)	Potent ROS and ferroptosis, synergistically improved therapeutic efficacy, low systemic toxicity achieved both in vitro and in vivo.	[86]
Cisplatin + Fisetin	Liposomes (173 nm)	Both drugs incorporated to a liposomal formulation composed by DOPC/cholesterol/DODA-GLY-PEG2000	U87 cell line	Additive effect of cisplatin and fisetin. Effective antitumoural action against GB cells.	[176]
Cisplatin + TMZ	Biomimetic NPs (171 nm)	pH degradable acetal grated dextran inner core coated with GB cancer cell membrane	U87 or drug-resistant U251R GB tumours (intravenous injection)	Improvement in BBB penetration and targeting of GB tissue/cells. Potent anti-GB activity in mice bearing orthotopic U87 or TMZ resistant U251 (U251R) tumours.	[177]
Carboplatin	Polymeric NPs	Polymer poly (ε-caprolactone) NPs	U87 MG	Increase uptake and cytotoxicity in U87 human GB cell line. No haemolytic activity in rat erythrocytes.	[186]
Carboplatin	Polymeric NPs (20–100 nm)	Polymer poly (ε-caprolactone) NPs	LN229 human GB cell line	Improved in vitro anti-tumour activity in comparison to free Carboplatin. Better nasal absorption compared to pure carboplatin solution and no severe damage of nasal mucosa.	[190]
Carboplatin	Polymeric NPs (365 nm)	Carboplatin-loaded poly (butyl cyanoacrylate) (PBCA) NPs conjugated with monoclonal antibody	C6 glioma cell lines C6 glioma-bearing rats (intraperitoneal injection)	Augmented cytotoxicity compared to free carboplatin. In vivo studies demonstrated an increased survival time and less side effects in the brain, kidney, or liver, compared to free carboplatin.	[187]
Carboplatin	Polymeric NPs (200 nm)	PLGA NPs	Primary rat hippocampal cell cultures. Adult male Wistar rats and white pigs (convection-enhanced delivery)	Improved cytotoxicity in vitro. Negligible neurotoxic effects and prolonged tissue half-life in vivo after infusion by CED in small and big animal models.	[188]
Oxaliplatin	Polymeric NPs (20–30 nm)	PEG-P(Glu) NPs functionalized with cyclic Arg-Gly-Asp (cRGD)	U87MG cell line	Target GB cells and ability to cross BBB. Tumour growth inhibition in orthotopic model.	[192]
Oxaliplatin	Liposomes (57–86 nm)	Liposomal formulation (Lipoxal^TM^)	F98 cell line (convection-enhanced delivery)	Administered by CED, increased tumoural accumulation, and median survival time in F98 glioma-bearing rats.	[193]
Oxaliplatin	CNTs (20 nm–several μm)	Multi-walled carbon nanotube functionalized with TAT/Biotin	C6 and CHEM-5 cell lines, C6 orthotopic glioma (intravenous injection)	Improved cytotoxicity of oxaliplatin toward glioma cells. Enhanced BBB penetration, improved brain-targeting selectivity, and excellent anti-tumour activity against orthotopic glioma.	[194]
Pt(IV) prodrug	Polymeric NPs (50–145 nm)	Cisplatin Pt(IV) prodrug in PLGA–PEG NPs	Canine J3TBG glioma and SDT3G glioblastoma cell lines (intravenous injection)	Overall 17 times more effective than cisplatin in vitro. Capable of crossing the canine BBB and accumulating in the brain.	[128]
Pt(IV) prodrug	CNTs (20 kDa ~ 4 nm)	Coil nanotubes	U87, U251, and patient GB cells, human astrocytes, U87 xenografts (subcutaneous injection)	Enhanced in vitro and in vivo tumour cell killing in comparison with free prodrug, activating multiple cell death pathways in GB cells without affecting astrocytes in vitro or causing damage to healthy mouse brain.	[93]
Pt(IV) prodrug	Polymeric NPs (105 nm)	Oxaliplatin-derived Pt(IV) complexes attached to a reduction-responsive polymer, (poly(CHTA-co-HD)-PEG)	U87, TMZ-resistant LN229 cell lines and cells from a patient-derived xenograft. In vivo LN229-TR xenografts (convection-enhanced delivery)	Improved cell uptake and cytotoxicity in comparison to TMZ. Lack of toxicity, favorable distribution of drugs into the region of interest, substantial tumour growth inhibition, and increased by 3 the survival time in mice bearing tumours after CED administration in vivo.	[197]
Pt(IV) prodrugs	Solid lipid NPs (SLNs) (30–80 nm)	Pt(IV) prodrugs derived from kiteplatin in (SLNs)	U87 human GB cell line, hCMEC/D3 endothelial cells	Enhanced permeability, improved cell uptake compared to free drug.	[198]
Pt(IV) prodrugs	Nanostructured Coordination Polymers (NCPs) (70 nm)	Pt(IV) prodrugs obtained from cisplatin as building block in NCPs	HeLa and GL261 cell lines. Orthotopic preclinical GL261 GB tumour-bearing mice (intranasal administration)	In vitro dual pH and redox-mediated control release. Comparable cytotoxicity to cisplatin. In vivo intranasal administration demonstrated increased tumour accumulation of platinum and effective anticancer action.	[199]
Pt^0^ NPs	Metal NPs (2–20 nm)	FePt NPs	U251, U87, and H4 cell lines	Marked cytotoxicity observed in lipophilic coated FePt NPs and low cytotoxicity in the case of hydrophilic FePt NPs.	[200]
Pt(0)	Metal NPs (42 nm)	Ag-Pt NPs	U87 cell line	Inhibition of Gram-positive and Gram-negative multi-drug resistant bacteria. Selective and dose-dependent anticancer activity over U87 GB cells without being harmful to healthy human fibroblasts.	[201]

Some references concerning use of metal-based Pt nanoparticles for GB treatment can also be found in literature. Thus, Kutwin et al. reported a comparative study between Pt-NPs and cisplatin against U87 GB cells or GB tumours growing on chorioallantoic membranes, observing that NPs showed antiproliferative activity although it was significantly lower than cisplatin [200]. Additionally, Lopez Ruiz et al. reported AgPt NPs with a selective and dose-dependent anticancer activity on human U87 GB and A375 melanoma cell lines (10–250 μg/mL concentration range) without compromising the activity of healthy human fibroblasts [201].

Concerning the best route of administration for nanoformulations containing platinum-based drugs, Paquette et al. published a series of papers combining Pt-based complexes and radiotherapy while assessing different administration routes, being the best results achieved with intra-arterial administration [104]. The same authors had already reported in 2012 cisplatin (lipoplatin) or oxaliplatin (lipoxal) liposomes administered by intracarotid infusion in orthotopic F98 glioma-bearing rats, obtaining notable results related to reduced toxicity, improved cell uptake, and increased survival of animals when combined with radiotherapy [97]. Afterwards, they also reported the combination of Pt drugs (administered via CED) and radiation treatment in the same murine model, increasing tumour drug retention, reducing side effects, and achieving a larger median survival time [202]. The combination of cisplatin-bearing AuNPs with radiotherapy was also used in in vitro resistant conditions to enhance DNA double-strand rupture and therefore apoptosis rate [203].

## 6. Why Repurpose Platinum Drugs and Use Nanotechnology?

Treatment of GB has remained almost unchanged for more than 20 years despite of continuous research efforts towards improvement. In the search of new treatment, platinum-based drugs have resurfaced as suitable alternatives for GB therapy. Although the developed anticancer platinum drugs are being used successfully to treat a variety of cancer types, most clinical assays with GB patients fail, in general due to dose-limiting toxicities when delivered systemically or in the tumour region, the low amount of drug doses that crosses the BBB, and/or systemic toxicities occurring before effective drug concentrations can reach tumours. However, the relative success of platinum drugs for treatment of non-CNS cancers suggests that there is hope regarding their therapeutic potential, provided they are properly delivered to the tumour region. Moreover, additional effects of platinum drugs such as immunomodulatory effects, and the use of specific delivery strategies that can maximize these multimodal effects and minimize toxicities, may help to foster their re-purposing. Early clinical trials with these agents offered great promise by using cisplatin [204], carboplatin [205], or oxaliplatin [206,207]. However, in most of the clinical trials, the combination of platinum drugs with radiation therapy and/or other chemotherapeutic agents showed no significant survival advantage. In addition, systemic and dose-limiting toxicity remains a key challenge related to the administration of platinum chemotherapeutics, including nanoformulations such as LipoplatinTM which is currently in a Phase III clinical trial for treatment of non-small cell lung cancer.

The DNA damaging effect of platinum-based drugs affects pathways related to cell invasion, angiogenesis, chemo- and radio-sensitization, and immunomodulation. A lower sustained platinum drug dose over longer times permits extended cell viability and reorganization of complex cellular pathways and the tumour microenvironment. Platinum compounds can considerably reduce the ability of GB cells to invade the surrounding tissue by downregulating matrix metalloproteinase (MMP) expression [208]; this is one of the key advantages of repurposing platinum drugs for GB therapy. Moreover, platinum compounds may have anti-angiogenic effects [41] and can enhance the efficacy of the current adjuvant therapies for GB (i.e., TMZ and radiotherapy) [42,43].

Additionally, it has been demonstrated that platinum drug-related immunomodulation can have an incidence in reversing GB-mediated immune evasion [209]. Moreover, platinum drugs are capable of modulating immunosuppressive features associated with numerous cancers including GB [210,211] and could even alter the profile of circulating immune cells or tumour-infiltrating immune cells [212]. Specifically, it has been demonstrated that oxaliplatin can induce immunogenic cell death, enhancing antitumour adaptive immune responses against antigens expressed by the dead cells [213]. However, cisplatin does not seem to induce immunogenic cell death [211]. Thus, future studies would be needed in order to investigate which platinum drugs are capable of modulating the glioma microenvironment, and to which extent, to improve the outcome of cancer treatments.

Apart from the intrinsic effect of the platinum complexes, the use of nanoformulated systems may also foster improvement of the therapeutic outcomes. As it has been shown through different examples along this review, the integration of platinum drugs and prodrugs in a nanocarrier permits to protect the active compound and increase its delivery to the desired sites. Moreover, it can improve platinum-based therapies by (i) increasing the solubility of the complexes and their blood half-life, (ii) reducing side effects through targeted delivery and broader tissue distribution, (iii) providing a sustained drug release, and (iv) allowing simultaneous incorporation and delivery of other anticancer drugs for combination therapy [214].

## 7. Summary and Future Perspectives

The scarce advancements in GB therapy and lack of efficient therapeutic alternatives makes this disease a relevant field for pushing forward cutting-edge research. In this regard, nanoencapsulation can offer a new dimension for the assessment of both novel drugs and classic compounds with poor BBB penetration and/or previous failure in clinical trials. Among the already known drugs with demonstrated anticancer activity, but not included as usual therapeutic option at GB relapsing, platinum complexes have properties that make them especially suitable for the development of novel nanotechnology-based therapeutics. Cisplatin is usually chosen as frontline treatment [35,173], while carboplatin is rather used at tumour recurrence [215]. Recent studies using platinum drugs or prodrugs, alone or combining cisplatin with other adjuvants, have proven promising improvement in survival rates. Overall, these factors might place the platinum complexes back in the spotlight to test the new pharmacokinetic advantage paradigms provided by the nanoparticle technology. However, even though successful preclinical studies have already been reported, no clinical phases have been reached, preventing products to reach the market.

The previously unrecognized or underestimated potential of platinum-based treatments can now offer another opportunity for platinum complexes. By using the principles of nanomedicine and chemistry formulation, new approaches for GB treatment can be developed, and hopefully reach clinical phases in the future. NPs can overcome some of the drug delivery limitations posed by the BBB, providing a controlled and sustained drug release in the site of action, targeting tumour cells, and reducing toxicity. Worth to mention, immunomodulation might be included in the newly recognized therapeutic effects, which may have broad application in future combination therapies for GB. In recent years, immunotherapy strategies have revolutionized the treatment of many cancer types, also increasing the hope for GB therapy. However, mostly due to the multifactorial immunosuppression occurring in the GB microenvironment, the poor knowledge of the neuroimmune system, and the presence of the BBB, the efficacy of immunotherapy in GB is still limited. New and recent strategies for GB treatment have employed immunotherapy combinations and have provided encouraging results in both preclinical and clinical studies. The lessons learned from clinical trials highlight the importance of tackling different arms of immunity.

Another interesting aspect lies in the investigation of novel non-invasive administration routes such as intranasal delivery, as alternatives to classical routes (i.e., CED or intravenous administration). Non-invasive brain targeting through the intranasal route arises as an attractive modality to circumvent the BBB along the trigeminal/olfactory neuronal pathways, thereby enhancing the therapeutic ratio with minimal systemic exposure [216,217]. The main goal of such novel administration routes is to provide effective drug concentration in brain tumours using reduced doses, overcoming the BBB while avoiding most of the systemic side effects. In other words, it is not a matter of how much Pt reaches the tumour site, but ensuring that enough Pt reaches the tumour milieu in order to trigger immunogenic cell damage and elicit host immune system. Last, but not least, it is also relevant to avoid systemic effects that are a limiting factor for single and combined Pt treatments for GB.

Additionally, besides the therapeutic use of Pt complexes as single drugs, the combination of Pt-based nanoparticles with other approaches such as chemotherapy, radiotherapy, immunotherapy, gene therapy, or functional inhibitors seems to be attractive and raise interesting possibilities. In this sense, it is important to highlight the preclinical evidence regarding combination immunotherapy in terms of both immune and survival benefits for GB management. Recent studies assessing the combination of different classes of immunotherapeutic agents (e.g., immune checkpoint blockade and vaccines) offer interesting perspectives that may be an inspiring starting point to develop future strategies facilitating the clinical translation to address the unmet medical needs of GB treatment.

## Data Availability

No new data were created.
